# Stress and wellbeing of junior doctors in Australia: a comparison with American doctors and population norms

**DOI:** 10.1186/s12909-016-0693-2

**Published:** 2016-07-19

**Authors:** Deanne S. Soares, Lewis Chan

**Affiliations:** Department of Urology, Concord Repatriation General Hospital, Sydney, Australia

## Abstract

**Background:**

Stress in doctors adversely affects decision-making, memory, information-recall and attention, thereby negatively impacting upon the provision of safe and high quality patient care. As such, stress in doctors has been subject to increasing scientific scrutiny and has amassed greater public awareness in recent years. The aims of this study are to describe stress levels and the psychological wellbeing of current junior medical officers (JMOs), and to compare this to their predecessors, American surgical residents and population norms.

**Methods:**

Post graduate years 1 & 2 doctors at a single metropolitan tertiary referral center were surveyed in 2009 and 2014 using two reliable and validated psychometric questionnaires, the Short Form-36 (SF36) and Perceived Stress Scale-14 (PSS14), with additional questions pertaining to demographics and training. The results were compared with published data from American general surgical residents and Australian age-matched population norms.

**Results:**

Mean stress levels were lower in 2014 (23 ± 7.2) than in 2009 (27.2 ± 7.6) (*p* = 0.017). The mean PSS-14 score was lower than that of American surgical residents, both before (26.8 ± 7.3, *p* = 0.003) and after (26.7 ± 8.2, *p* = 0.004) implementation of the safe working hour policies but higher than societal controls (*p* < 0.0001). Whilst JMOs in 2014 reported better overall mental health compared to those in 2009 (*p* = 0.02), they were significantly worse than the general population (*p* = 0.009). Multivariate analysis showed that JMOs were more likely to have a high PSS-14 score or to have a low mental health score if they reported higher career anxiety (*p* < 0.05).

**Conclusions:**

Doctors are still at risk despite an improvement in their stress levels and overall mental health. They are less likely to be stressed and to have better mental health if they have less career-related anxiety. This has implications for the medical education and training of our junior doctors.

## Background

The challenges of first and second postgraduate years (PGY 1 & 2) of medical training are unique and numerous. This period of transition from student to the dual role of learner and healthcare provider can therefore be very stressful, given the increased responsibility and expectations. Several studies have identified higher rates of fatigue, distress, burnout, anxiety and depression amongst doctors [1–5]. Stressors identified have been categorised into situational (e.g. increased workload, sleep deprivation, conflict), professional (e.g. high levels of responsibility, career planning) and personal (e.g. financial debt, inadequate coping skills, unhealthy lifestyle) [4–7]. In addition, other stressors specific to this transitional period have been noted such as difficulty with application of knowledge, uncertainty of expectations, increased on-call responsibilities and experiencing the death of patients [8, 9]. High stress and burnout has the potential to negatively impact upon work performance and patient care, including medication errors, suboptimal care, clinical errors and patient dissatisfaction [6, 10, 11]. Despite this, only a few studies have been done to assess the level of stress and the overall health and wellbeing of PGY1&2 doctors in Australia [12–14] and importantly, none make any comparisons to other countries. The aim of this study is to describe the extent of stress in current junior doctors and their psychological well being in comparison to their predecessors, American surgical residents and Australian population norms. Additionally, this study also aims to identify socio-demographic or professional factors that influence these. The rationale for this study was that by understanding the current state of junior doctors and the factors that impact their wellbeing, educators, trainers and hospitals could better service the needs of PGY1 & 2 doctors in Australia.

## Methods

### Participants

The two cohorts consisted of PGY 1 & 2 doctors working at Concord Repatriation General Hospital, a major metropolitan hospital, in 2009 and 2014. Written informed consent for participation in the study was obtained from participants.

### Design

A survey of trainees was undertaken at the end of the clinical year at two points over a five-year period. Data was collected at the end of the clinical year so as to minimise any potential bias due to upcoming examinations or a new clinical rotation. Participation was voluntary and anonymous. Two validated questionnaires, the Short Form 36 Health Questionnaire (SF36) [15] and the Perceived Stress Scale 14 (PSS14) [16], were used to assess the general wellbeing and stress amongst participants. Demographic data sought included variables such as age, gender, marital status, number of children and training related variables such as location and satisfaction with current posting. The results from the PSS14 were compared to published data from American surgical residents before and after the introduction of the 80 h per week restrictions as well as to societal historical controls [17, 18]. The results from the SF-36 were compared with results from a national population study published by the NHMRC in 1995 [19]. The study was approved by the Human Research Ethics Committee, Concord Repatriation General Hospital.

### Measurement tools

#### Perceived stress scale 14 (PSS14)

This is a 14 item questionnaire published by Cohen et al. in 1983, designed to measure the degree to which situations in one’s life are appraised as stressful [16]. This questionnaire has been widely used as a reliable and valid tool for measuring perceived stress levels in a population group [16]. The questions were designed to gauge how uncontrollable, unpredictable and overloaded the participants find their lives [16]. It is predictive of objective biological markers of stress and increased risk for certain diseases in those with higher perceived stress levels. The PSS-14 was designed to use on those with at least a junior high school education. Each of the 14 items is scored from 0 to 4 with a total score range between zero and 56. A high score correlates with a high perception of stress [16].

#### Short form 36 health questionnaire (SF-36)

The SF36 is a survey that was designed to provide information about the general health and wellbeing of the participant [15]. This survey is a concise version derived from a larger set of questions used in the Medical Outcomes Study in 1989 [20]. It has been demonstrated to produce reliable and valid results in both the clinical and population setting and has been used internationally [15, 19]. The SF-36 provides indicators across eight dimensions:

- **Physical functioning:** Indicative of the extent of limitation in performing daily activities due to the individual’s health.
- **Bodily pain:** Indicative of severity of pain and interferences with regular activities.
- **General health perceptions:** Indicative of the participants’ current health status and their perceptions of this compared to the health status of other individuals.
- **Physical role functioning:** Indicative of the impact of the individual’s physical health on their work performance or other activities.
- **Emotional role functioning:** Indicative of the impact of the individual’s emotional problems on their work or other activities.
- **Social role functioning:** Indicative of the impact of the individual’s physical or emotional status on their social activities.
- **Mental health:** Indicative of the individual’s experiences of anxiety, depression, nervousness and happiness.
- **Vitality:** Indicative of the individual’s energy level or fatigue.

Each question contributes to a score for each dimension. The values of each response category are added across contributing questions and then expressed as a score from 0 to 100 for each of the eight dimensions. A higher score indicates a better state of health or wellbeing. All the dimensions, expect for physical functioning and general health, are focussed on the participants health and wellbeing in the 4 weeks prior to when the survey is done [19]. There are also two summary scales- the Physical Component Summary (PCS) and the Mental Component Summary (MCS) which are derived from the scores using standardised populations data [19].

### Statistical analysis

Descriptive statistics were used for the research variables and Student’s *t*-test for comparisons between the means of continuous variables. Multiple logistic regression analysis was performed in order to evaluate the contributing factors to outcome measures of perceived stress and wellbeing of participants. The tests were all 2-tailed and the P value for statistical significance was set at 0.05. All statistical analysis was performed using SPSS software for Mac, version 21.0.

## Results

38 of 46 (82 %) PGY 1 & 2 doctors responded to the survey in 2014 compared to 79 % in 2009. 60 % of the respondents were female with the majority in both sexes being less than 28 years old. Over 85 % of the participants in both cohorts did not have any children and a large majority of those surveyed were in a metropolitan posting at the time. The socio-demographic characteristics of the participant groups are given in Table 1. The gender and age distribution was not significantly different between the two groups.

**Table 1:** Socio-demographic characteristics of participants

	2009	2014
Characteristic	n (%)	n (%)
Gender		
Male	14 (40)	16 (42.1)
Female	21 (60)	22 (57.9)
Age (y)		
< 28	17 (48.6)	20 (52.6)
28-32	9 (25.7)	15 (39.5)
> 32	9 (25.7)	3 (7.9)
Number of children		
0	31 (88.6)	33 (86.6)
1	4 (11.4)	5 (13.2)
2 or more	0 (0)	0 (0)
Clinical posting		
Metropolitan	35 (100)	29 (76.3)
Rural	0 (0)	9 (23.7)

### Professional variables

76.3 % of the 2014 cohort rated the academic value of their clinical rotation as good or very good. This was better than the 2009 cohort, with only 40 % rating the academic value that highly (*p* = 0.002). Similarly, significantly more doctors in 2014 (76.3 %) rated the practical value of their clinical post to be good or very good than those in 2009 (42.9 %)(*p* = 0.004). More doctors in 2014 also rated the overall enjoyment of their current clinical post to be better than average (81.6 % vs 51.4 %, *p* = 0.006). See Table 2.

**Table 2:** Junior doctors’ ratings of current clinical post with regards to academic value, practical value and overall enjoyment

	2009	2014
	n (%)	n (%)
Academic learning value		
Poor	1 (2.9)	1 (2.6)
Deficient	6 (17.1)	1 (2.6)
Average	14 (40)	7 (18.4)
Good	10 (28.6)	20 (52.6)
Very good	4 (11.4)	9 (23.7)
Practical learning value		
Poor	1 (2.9)	1 (2.6)
Deficient	6 (17.1)	2 (5.3)
Average	13 (37.1)	6 (15.8)
Good	6 (17.1)	15 (39.5)
Very good	9 (25.7)	24 (36.8)
Overall enjoyment		
Poor	2 (5.7)	1 (2.6)
Deficient	5 (14.3)	3 (7.9)
Average	10 (28.6)	3 (7.9)
Good	13 (37.1)	21 (55.3)
Very good	5 (14.3)	10 (26.3)

### Career anxiety

**Fig. 1:**
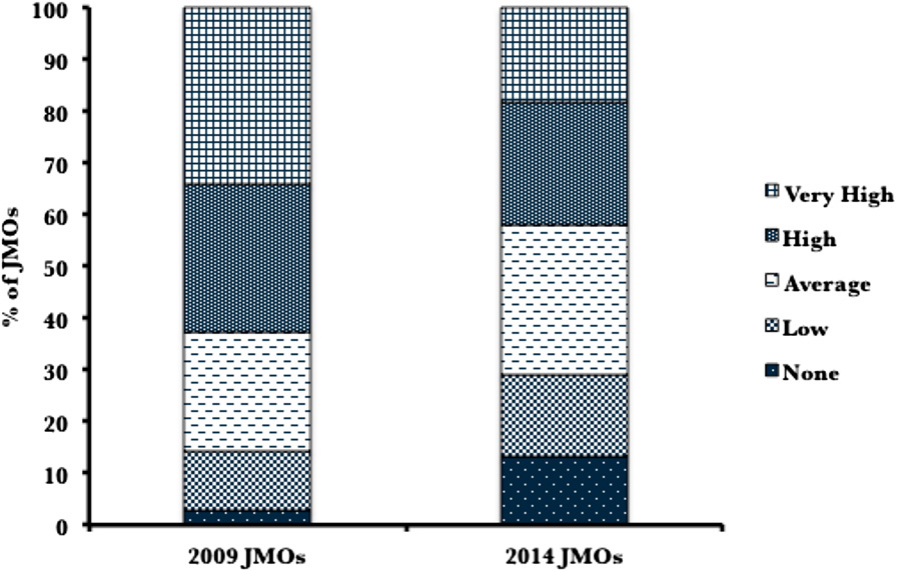
Self reported career anxiety. Participants were asked how often they felt anxious about their future career path

The proportion of doctors reporting high anxiety with regards to their future career paths has decreased over the last 5 years but this did not reach statistical significance. Career anxiety was rated to be high or very high by 42.1 % in 2014 compared to 62.9 % in 2009 (*p* = 0.07). See Fig. 1.

### Perceived stress

**Fig. 2:**
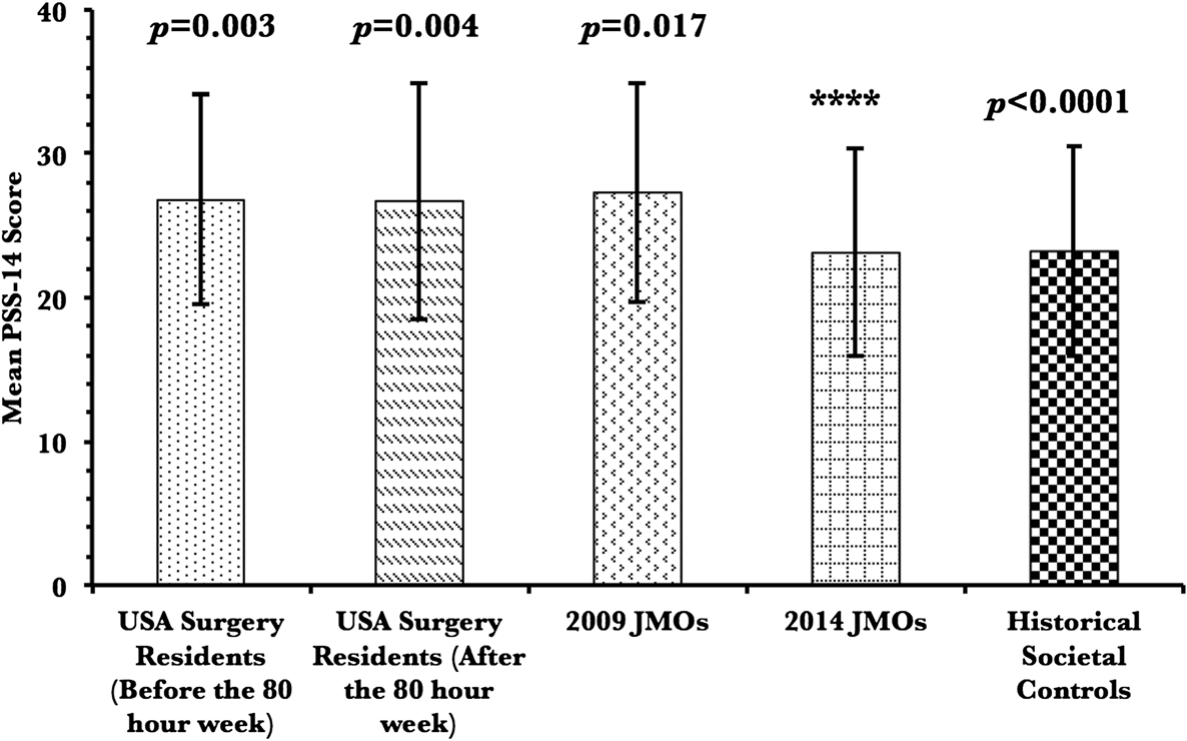
Outcome measure for perceived stress. Mean PSS-14 scores are shown for JMOs in 2009 and 2014 as well as for surgical residents in the USA before and after the implementation of the 80 h week. *P* value compared to 2014 cohort given above each group

The mean perceived stress score among junior doctors in 2014 (23.1 ± 7.2) was lower than in the 2009 cohort (27.3 ± 7.6) (*p* = 0.017). It was also lower than the mean PSS-14 scores of surgical residents in America both before (26.8 ± 7.3, *p* = 0.003) and after (26.7 ± 8.2, *p* = 0.004) the implementation of safe working hour policies in the USA. However, when comparing the mean PSS-14 scores to published historical normative data, junior doctors in both cohorts were found to have significantly higher scores (*p* < 0.0001). See Fig. 2.

### Health and wellbeing

Emotional role functioning and mental health were poorer in the 2009 cohort compared to the 2014 cohort. There was no statistical difference amongst the 2 groups, with any of the other health and wellbeing dimensions. However, general health perceptions (*p* = 0.001), vitality (*p* < 0.0001) and social role functioning (*p* = 0.001) were considerably worse in junior doctors compared to the general population. Whilst the overall mental health of the 2014 cohort was better than that of the 2009 cohort, it was significantly worse than the general population. See Table 3.

**Table 3:** SF 36 scores for 2014 junior doctors and comparisons with previous cohort and population norms

Dimension	2014 cohort mean score	p value vs 2009 cohort	p value vs population norms
Physical functioning	91.71 ± 17.75	0.831	0.677
Physical role functioning	84.86 ± 34.65	0.515	0.773
Bodily pain	80.82 ± 17.66	0.945	0.813
General health perceptions	64.24 ± 19.83	0.721	0.001^b^
Vitality	46.58 ± 19.67	0.265	0.000^c^
Social role functioning	72.70 ± 22.11	0.914	0.001^b^
Emotional role functioning	85.96 ± 32.54	0.037^a^	0.970
Mental health	71.58 ± 15.39	0.003^b^	0.072
Physical Component Summary	53.31 ± 7.64	0.259	0.865
Mental Component Summary	44.95 ± 9.54	0.02^a^	0.009^b^

^a^Correlation is significant at the .05 level (2-tailed)

^b^Correlation is significant at the .01 level (2-tailed)

^c^Correlation is significant at the 0.001 level (2-tailed)

### Correlation of perceived stress and wellbeing with personal and professional variables

Multivariate regression analysis did not show any significant association between age, gender, number of dependents or location of posting to perceived stress levels or mental health in junior doctors. However, they were more likely to have a high levels of stress or poor mental health if they reported high anxiety related to their career (*p* < 0.05). They were also likely to have poorer mental health if they did not find they current clinical posting to be enjoyable or to be poor in academic or practical learning value (*p* < 0.05).

## Discussion

Data on the health and wellbeing of junior doctors in Australia is limited and as such this study provides pertinent information in this area of interest. This study was designed to minimise any potential bias with the use of reliable and validated questionnaires [15, 16]. Additionally, the surveys were conducted at the end of the clinical year so as to reduce the potential impact of stressors such as new clinical rotations, examinations and traditionally high-stress periods.

We found that overall, the current cohort of junior doctors are more satisfied with their clinical posting than their predecessors and found it to be useful in academic and practical learning value. The 2014 cohort of PGY 1&2 doctors are also less stressed than their predecessors as well as compared to American surgical residents. This was both before and after the implementation of the 80 h week in the USA [17, 18]. We currently do not have any legislated work-hour restrictions in Australia and the lower levels of stress may be a consequence of other differences between the Australian and American healthcare system. Both the Australian and American health care environments are complex and have several obvious differences between the two systems. Some of these differences are with governance and administration, professional roles, work hours of medical personnel, duration of undergraduate and graduate education, financial debt of doctors and competitiveness of residency programs [21]. Any or all of these could have contributed to the results seen in this study. However, this study was not designed to assess these differences and we can only make assumptions about the contributory effect of these factors. We compared our result to American surgical trainees because of the use of the same measuring tool. One of the difficulties of a meaningful comparison with other studies is the use of many different scales and questionnaires in order to ascertain stress levels in the participating group. It is important to note that the use of SF-36 in our study allows us to make a direct comparison to American surgical residents [17, 18] and whilst fraught with challenges, is the only study to do so.

Despite the decreasing levels of stress in our junior doctors, they were found to be more stressed than the general population and are therefore, still at risk. Numerous other studies have also found high rates of stress and burnout in junior doctors. Cohen and Patten [22] found 34 % of resident doctors in Alberta, Canada to be stressed. Another study by Sargent et al. [23] found similar rates of stress prevalence in the USA. Other studies have also found higher rates of anxiety and depression in doctors compared to the general population [6, 10, 11]. Poorer wellbeing and mental health in doctors has been found not only in different countries around the world [24, 25] but also in different fields within medicine [6, 8, 22]. Factors that have been implicated have been sleep deprivation, long work hours, debt, fatigue and career anxiety [1–7].

We found that there was a significant correlation between stress and the mental health of doctors and the anxiety they experience with regards to their career and overall clinical enjoyment. Interestingly though, no correlation was found between stress level and/or health and wellbeing scores and factors such as age, gender and number of dependents. When analysing the overall health and wellbeing of junior doctors in the two cohorts, there were minor improvements in some of the dimensions tested, including mental health. Yet, there was still a difference between the current cohort and the general population, with our doctors reporting poorer general health, vitality, social functioning and overall mental health.

The link between poor mental health and high career anxiety is an interesting one. There has been a significant rise in the number of both domestic and international medical graduates recently. There has been a 35 % increase of domestic students from 2004 to 2008 and 62.4 % rise from 2009 to 2014 [26]. These increases have not been mirrored in vocational trainee numbers [26] and this could be a reason for ongoing career anxiety amongst junior doctors. Although given that our 2014 cohort reported less career-related anxiety than the 2009 cohort despite this increase in graduating doctors, there must be other factors that are contributing to their career-related anxiety. Perhaps doctors harbour personalities that are more prone to anxiety and stress compared to the general population. In fact, one study showed that two personality traits, reality weakness and neuroticism, predicted mental health problems amongst junior doctors [27]. However, this does not fully explain why junior doctors are particularly vulnerable to poor mental health and high levels of stress. In fact, in the 2013 National Mental Health Survey of Doctors and Medical Students, younger doctors reported high rates of emotional exhaustion, low professional efficacy and high cynicism causing higher levels of burnout than in older doctors [28]. This is highly suggestive of issues with the process of transition and the requirement for increased support during this time. Our study also suggests that the clinical environment, particularly certain aspects of training, plays an important role in the mental health and emotional wellbeing of junior doctors in Australia. This concept is supported by another study [7], in which doctor wellbeing improved when they perceived high team support and experienced valued learning opportunities.

The focus of previous interventions to reduce stress and improve the wellbeing of doctors has been on personal and individual strategies such as stress reduction, mindfulness and grief training [7]. However, given the findings of our study and similar other studies [1–7], we suggest the need to have interventions at an organizational level in order to address the training, curricular and system factors that seemingly contribute to the mental health of junior doctors. One of the paramount goals of this study was to provide an insight into the current health and wellbeing status of Australian junior doctors and to thus stimulate additional focused research. This can provide impetus for further exploration and re-evaluation of our current clinical environment, education and training in order to ensure strong development of the profession as a whole.

### Limitations

When considering aspects of the methodology it is important to note that all the variables in this study are based on self-assessment and this could have implications on the relevance of the information provided. We did however use two widely used and reliable measurement tools with proven reliability. Also, this study was done on a small cohort of participants at a single institution and this may affect the generalisability of the results to all junior doctors in Australia. A larger cohort across different setting should be utilized in future studies in order to avoid potential selection bias. We are also unable to definitively exclude a non-response bias as individuals with high levels of stress or poor mental health may have been less inclined to participate in this study. However, our high participation rate suggests that most respondents were willing to partake in this study and we therefore expect this bias to be low.

## Conclusion

This study contributes to the examination of the stress experienced by junior doctors in Australia and gives us an insight into their health and wellbeing. Stress levels have improved in recent times and there have also been some improvements in their emotional wellbeing. However, their stress levels are still worse than societal controls. Additionally, aspects of their general health and wellbeing are certainly not at the same level as age-matched Australian population norms, which is also cause for concern. Interestingly, no association was found between perceived stress levels or mental health status and socio-demographic factors such as age, gender or number of dependents. Rather, our junior doctors are less likely to be stressed and have overall better mental health if they are less anxious with regards to their career, enjoy their work and perceive it to be high in educational value. This suggests that providing good training and creating a suitable learning environment can improve doctor wellbeing and could therefore potentially have a positive impact upon patient care.

## Abbreviations

JMO, Junior Medical Officer; MCS, mental component summary; PCS, physical component summary; PGY1, post graduate year 1; PGY2, post graduate year 2; PSS14, perceived stress scale-14; SF36, short form 36

## References

[1] Thomas NK (2004). Resident burnout. JAMA.

[2] Hurst C, Kahan D, Ruetalo M, Edwards S (2013). A year in transition: a qualitative study examining the trajectory of first year residents’ well-being. BMC Med Educ.

[3] Gundersen L (2001). Physician burnout. Ann Intern Med.

[4] Lefebvre D (2012). Perspective: resident physician wellness: A new hope. Acad Med.

[5] Rosen IM, Gimotty PA, Shea JA, Bellini LM (2006). Evolution of sleep quantity, sleep deprivation, mood disturbances, empathy, and burnout among interns. Acad Med.

[6] Shanafelt TD, Bradley KA, Wipf JE, Back AL (2002). Burnout and self-reported patient care in an internal medicine residency program. Ann Intern Med.

[7] West C, Shanafelt T, Kolars J (2011). Quality of life, burnout, educational debt, and medical knowledge among internal medicine residents. JAMA.

[8] Tallentire VR, Smith SE, Skinner J, Cameron HS (2011). Understanding the behaviour of newly qualified doctors in acute care contexts. Med Educ.

[9] Brennan N, Corrigan O, Allard J (2010). The transition from medical student to junior doctor: today’s experiences of tomorrow’s doctors. Med Educ.

[10] West CP, Tan AD, Habermann TM, Sloan JA, Shanafelt TD (2009). Association of resident fatigue and distress with perceived medical errors. JAMA.

[11] Fahrenkopf AM, Sectish TC, Barger LK (2008). Rates of medication errors among depressed and burnt out residents: prospective cohort study. BMJ.

[12] Markwell AL, Wainer Z (2009). The Health and wellbeing of junior doctors: insights from a national survey. Med J Aust.

[13] Willcock SM, Daly MG, Tennant CC, Allard BJ (2004). Burnout and psychiatric morbidity in new medical graduates. Med J Aust.

[14] Schattner P, Davidson S, Serry N (2004). Doctors’ health and wellbeing: taking up the challenge in Australia. Med J Aust.

[15] Ware JE, Gandek B, Sinclair SJ, Kosinski M (2004). Measuring and Improving Health Outcomes: An SF-36® Primer for the Medicare Health Outcomes Survey.

[16] Cohen S, Kamarck T, Mermelstein R (1983). A global measure of perceived stress. J Health Soc Behav.

[17] Zare SM, Galanko J, Behrns KE (2004). Psychological wellbeing of surgery residents before the 80-h work week: a multiinstitutional study. J Am Coll Surg.

[18] Zare SM, Galanko JA, Behrns KE (2005). Psychologic wellbeing of surgery residents after inception of the 80-h workweek: a multi-institutional study. Surgery.

[19] Australian Bureau of Statistics (1997). 1995 National Health Survey: SF-36 population norms, Australia. (Cat. No. 4399.0).

[20] Tarlov AR, Ware JE, Greenfield S, Nelson EC, Perrin E, Zubkoff M (1989). The Medical Outcomes Study: An application of methods for monitoring the results of medical care. JAMA.

[21] Jones PD, Seoane L, Deichmann R, Jr, Kantrow C. Differences and similarities in the practice of medicine between Australia and the United States of America: challenges and opportunities for The University of Queensland and the Ochsner Clinical School. Ochsner J. 2011 Fall; 11(3): 253-258. PMC3179196 21960759

[22] Cohen JS, Patten S (2005). Well-being in residency training: A survey examining resident physician satisfaction both within and outside of residency training and mental health in Alberta. BMC Med Educ.

[23] Sargent MC, Sotile W, Sotile MO, Rubash H, Barrack RL (2004). Stress and coping among orthopaedic surgery residents and faculty. J Bone Joint Surg Am.

[24] Lue B, Chen H, Wang C, Cheng Y, Chen M (2010). Stress, personal characteristics and burnout among first postgraduate year residents: a nationwide study in Taiwan. Med Teach.

[25] Al-Dubai S, Ganasegeran K, Perianayagam W, Rampal K (2013). Emotional burnout, perceived sources of job stress, professional fulfillment, and engagement among medical residents in Malaysia. Scientific World Journal.

[26] Department of Health | Health workforce [Online]. Health.gov.au. 2016 [cited 24 May 2016]. Available from: http://www.health.gov.au/internet/main/publishing.nsf/Content/Health+Workforce-2.

[27] Gramstad T, Gjestad R, Haver B (2013). Personality traits predict job stress, depression and anxiety among junior physicians. BMC Med Educ.

[28] Beyond Blue. National Mental Health Survey of Doctors and Medical Students [Online]. 2013 [cited 25 May 2016]. Available from: https://www.beyondblue.org.au/docs/default-source/research-project-files/bl1132-report---nmhdmss-full-report_web.

